# Human latent inhibition: Problems with the stimulus exposure effect

**DOI:** 10.3758/s13423-018-1455-4

**Published:** 2018-03-19

**Authors:** N. C. Byrom, R. M. Msetfi, R. A. Murphy

**Affiliations:** 1 Department of Psychology, Institute of Psychiatry, Psychology and Neurosciences, Guy’s Campus, Kings College London, London, UK; 2 Centre for Social Issues Research, Department of Psychology, University of Limerick, Limerick, Ireland; 3 Department of Experimental Psychology, University of Oxford, Radcliffe Observatory Quarter, Oxford, England

**Keywords:** Latent inhibition, Associative learning, Masking tasks, Priming

## Abstract

Latent inhibition (LI) is a startlingly simple effect in which preexposure of a stimulus without consequence retards subsequent responding to a stimulus–consequence relation. The effect was first demonstrated with Pavlovian conditioning in animals and was later suggested to be a marker of human psychopathology such as schizophrenia. Individual differences in LI has supported the continued use of animal models to understand human mental health. In this review, we ask whether there is sufficient evidence to support the continued application of LI from animal models to human psychopathology because of the weak evidence for LI in humans. There is considerable variability in the methods used to assess LI, sustaining different theoretical accounts of the effects observed, which differ from the accepted accounts of LI as demonstrated in animals. The review shows that although there have been many experiments testing human LI, none provide the necessary experimental controls to support the conclusion that retarded responding is caused simply by preexposure to a stimulus, as has been demonstrated with animal models. Establishing this conflict, we set out a framework for future research.

Learning from experience involves acquiring connections or associations between stimuli and outcomes in our environment. Such learning allows the memories associated with stimuli we encounter to guide actions and predict outcomes. For instance, if Jo drives the same route to work each day, the actions required to navigate the route safely become automatic, cued by familiar stimuli along the way. Through experience, Jo has learnt associations so that the stimuli in her environment can activate memories to prompt efficient instrumental responses. Learning these regularities so that stimulus–action memories are activated in different situations is adaptive, facilitating efficient responding to achieve motivated outcomes. However, while learnt associations help us respond when conditions are similar to the past, they can slow down our ability to form new associations and adapt to new situations; they can also make us less flexible.

One way to investigate cognitive and behavioral flexibility is to examine how prior experiences influence learning. In this review, we focus on the specific case of stimulus preexposure. Preexposing a stimulus in the absence of an outcome retards subsequent responding to the same stimulus if it is trained to be a predictor of an outcome (Lubow, 2010; Lubow & Moore, 1959). The effect, first labelled the CS preexposure effect and then the more theoretically laden label latent inhibition (LI), was reported first with laboratory animals (e.g., Baker & Mackintosh, 1979; Lubow, Markman, & Allen, 1968; Lubow & Moore, 1959; Rescorla, 1971) and, subsequently, with humans (e.g., Ginton, Urca, & Lubow, 1975). This effect of preexposure illustrates that previous regularities influence current behavior and demonstrates a limit to cognitive and behavioral flexibility. However, while there have been many tests of LI in humans, it is unclear whether they provide a simple measure of the effect of stimulus preexposure on current behavior. Through this review, we aim to evaluate the human LI research and ask what tests of human LI are measuring.

It is unclear what experimental tests of human LI are measuring. This question is particularly important in the context of continued interest in individual differences in the LI effect. The LI effect received considerable research interest following the observation of individual differences in human tests of LI (for further discussion, see Byrom & Murphy, 2018). Some individuals show a reduced LI effect, showing no effect of preexposure but rather adaptation of behavior to respond to a stimulus when a new stimulus–outcome association is trained. The absence of a preexposure effect has been described in individuals with acute schizophrenia (e.g., Baruch, Hemsley, & Gray, 1988a; but see also Schmidt-Hansen & Le Pelley, 2012), high levels of schizotypy (e.g., Lubow, Ingberg-Sachs, Zalstein-Orda, & Gewirtz, 1992; Baruch, Hemsley, & Gray, 1988b; Gray, Fernandez, Williams, Ruddle & Snowden, 2002), acute and chronic stress (Braunstein-Bercovitz, Dimentman-Ashkenazi, & Lubow, 2001; Braunstein-Bercovitz, Rammsayer, Gibbons, & Lubow, 2002), high levels of creativity (Burch, Hemsley, Pavelis, & Corr, 2006; Carson, Peterson, & Higgins, 2003), obsessive-compulsive disorder (Kaplan et al., 2006; Lee & Telch, 2010; Swerdlow, Hartston, & Hartman, 1999), and depression (Msetfi, Byrom, & Murphy, 2017).

Disruptions to LI have been interpreted as indicative of increased cognitive or behavioral flexibility (Burch et al., 2006) as well as reflecting a weakening of the earlier memory or shifting of attention and therefore a reduced ability to use past regularities to guide learning (e.g., Hemsley, 1993). From this perspective, in addition to providing insight into cognitive and behavioral flexibility, LI has the potential to provide a model of some components of psychological disturbance (Weiner & Arad, 2009). Indeed, the interest in individual differences in LI has contributed to the use of animal models of LI to develop neurodevelopmental understanding of schizophrenia (e.g., Jeevakumar et al., 2015; Meyer & Feldon, 2012; Piontkewitz, Arad, & Weiner, 2012; Vuillermot et al., 2012) and potential pharmacological interventions (e.g., Mizuno et al., 2013; Piontkewitz, Arad, & Weiner, 2011; Singer, Wei, Chen, Boison, & Yee, 2013; for a review, see Moser, Hitchcock, Listers, & Moran, 2000; Weiner & Arad, 2009). However, there is good reason to suggest that the nature of effect recorded in animals is qualitatively distinct from that observed in humans.

Researchers have begun to call into question the relationship between LI and schizophrenia (Schmidt-Hansen & Le Pelley, 2012) and the related trait measure of schizotypy (Granger, Moran, Buckley, & Haselgrove, 2016). These articles draw attention to replication failures, specifically the failures to find evidence of weakened LI in individuals with schizophrenia or high levels of schizotypy. Failures to replicate individual differences in LI could indicate that the effect in question is not “real.” However, replication failures can also indicate statistical and power issues, or a failure to follow previous procedure (Van Bavel, Mende-Siedlecki, Brady, & Reinero, 2016). Replication failures highlight the sensitivity of LI; changes in the methods used to test LI have had a substantive influence on the observation of the effect and the very nature of the effect being studied.

In the present review, we set out to consider the various methods used to test LI. First, however, we introduce a brief history of LI research to contextualize the main review.

## A brief history of latent inhibition

### Animal studies

At the time the first LI experiments were conducted, researchers were interested in the mechanism by which learning occurred in the absence of reinforcement (i.e., latent learning). Performance of a rat navigating a maze could be improved by providing reward (e.g., food). What was more difficult to explain was why prior exposure to the maze *without* reinforcement could sometimes enhance or sometimes suppress learning once a reward was introduced. The effects of prior exposure were explained in terms of latent learning. If learning following preexposure involved acquiring new responses to a stimulus, then responses acquired during the preexposure phase could suppress new learning to the extent that the two responses were in conflict (Thistlethwaite, 1951; Tolman, 1948) but enhance learning if they shared features.

In this context, Lubow and Moore (1959) measured the interference created by nonreinforced preexposure to a single stimulus, as is summarized in Table 1. They hypothesized that latent learning was a response-mediated effect and that preexposing a stimulus would allow for responses to that stimulus to be learnt that would interfere with the subsequent acquisition of new, alternate responses when the stimulus was paired with a biologically relevant outcome (unconditioned stimulus; US). Thus, they reasoned that the rate of conditioning (learning the new response) would be slower for a preexposed stimulus than for a non-preexposed stimulus. In line with their hypothesis, animals preexposed to a stimulus took more trials to reach a criterion level of conditioned responding than if the stimulus that had not been preexposed.

**Table 1:** Design of the Lubow and Moore (1959) study

Phase 1: Preexposure phase	Phase 2: Test phase	Number of trials to criterion
10 × PE CSt → no outcome	PE CSt → Outcome	26
	NPE CSt → Outcome	19

CSt = target conditioned stimulus; PE = preexposed; NPE = not preexposed

**Fig. 1:**
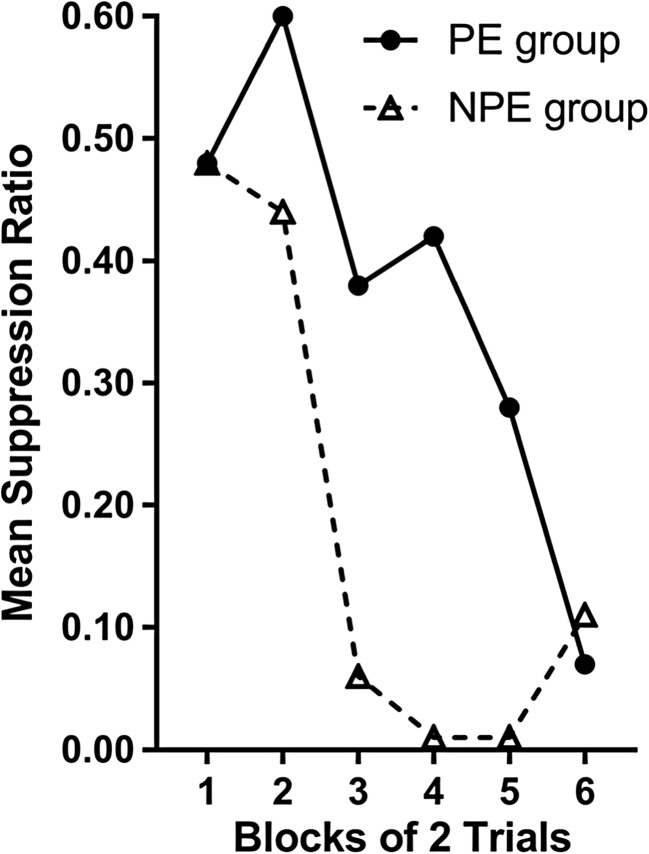
Data redrawn from Rescorla (1971), showing change in mean suppression ratio of Phase 2 trials for a preexposed and non-preexposed group

While Lubow and Moore (1959) measured the number of trials to a criterion as an index of interference, the LI effect can also be inferred by differences in the *rate* of change in behavior. For example, Rescorla (1971) trained two groups of rats to lever press for food (see Table 2). In Phase 1, the preexposed group was exposed to the to-be-conditioned stimulus (CSt) in the absence of an outcome, while the non-preexposed group had no exposure to the stimulus. In Phase 2, both groups received pairings of the conditioned stimulus (CSt) and the outcome (O; electric shock). Rescorla predicted that pairing the CSt with an aversive outcome would lead the animals to freeze in the presence of the CSt (immobility is a common behavior acquired to stimuli that precede aversive stimuli like shock), and this would be incompatible with other behaviors, like lever pressing for food pellets. As such, acquisition of the stimulus–outcome association could be assessed by measuring lever pressing in the presence of the CSt and comparing this to the rate of lever pressing in the absence of the CSt, as shown in Fig. 1. The suppression ratio (the ratio of the rate of lever pressing in the presence of the CSt with the rate of lever pressing in the absence and presence of the CSt) for all rats started at around .5 (i.e., the rats were lever pressing equally in the presence and absence of the CSt). Across Phase 2, the suppression ratio fell toward zero, as rats ceased to press the lever in the presence of the CSt. The rate of decline in the suppression ratio was slower for the preexposed group. By measuring the suppression ratio over trials, Rescorla showed the interference effect of preexposure on a trial-by-trial basis.

**Table 2:** Design of the Rescorla (1971) study

Group	Phase 1: Preexposure phase	Phase 2: Test phase
Preexposure group (PE)	CSt → no outcome	CSt → Outcome
No preexposure group (NPE)	/	

CSt = target conditioned stimulus

### Human studies

Latent inhibition has also been reported in human learning. Using a comparable form of conditioning, Schnur and Ksir (1969) observed an effect of preexposure on human eyelid conditioning. Participants were preexposed to a CSt prior to this being paired with an outcome (a puff of air delivered to the eye) and a conditioned response (eye blinks) being measured. Illustrating an LI effect, participants who were preexposed to the CSt were slower to acquire the conditioned response than were participants receiving no preexposure or preexposure to a different stimulus. This was all completed in the presence of a masking task; participants were led to believe that the study required them to press buttons in response to lights presented. The masking task had no relevance to the LI task, and participants were not aware that the study was assessing conditioned responses. Much of the research with humans has employed masking tasks during the preexposure phase nominally to engage the participants’ attention.

Subsequent human studies have described LI effects using protocols extending beyond conditioned responding for appetitive and aversive stimuli, to preparations involving cognitive processing for neutral stimuli (hence, we refer to stimuli in these experiments as S rather than CS). For example, Lubow et al. (1992) developed a visual learning task that has subsequently been used widely and adapted by many other labs (see “Visual” task in Table 3 for a summary of this design). In Phase 1, participants complete a masking task with visual trigrams presented successively. Participants are required to count the number of times a specific trigram occurs. For the preexposed (PE) group, the masking task stimuli are superimposed on the St (a shape). The non-preexposure (NPE) group completes the masking task with no exposure to the St. The stimuli from the masking task continue to be presented through Phase 2, during which all participants are presented with a discrimination task in which the St predicts an outcome (increments on a score counter) while a novel, nontarget stimulus (Snt) predicts no outcome. Participants are required to respond to prevent the outcome from occurring. As such, participants should respond in the presence of St and withhold responding in the presence of Snt. The dependent measure is the number of trials a participant takes to reach a criterion of five consecutive correct responses to St with no responses to Snt. Lubow et al. found that participants took longer to reach the response criterion if they had been preexposed to the St. Similar findings have been replicated in many subsequent studies (e.g., Lipp, 1999; Swerdlow, Braff, Hartston, Perry, & Geyer, 1996; Gray et al., 2001; Zalstein-Orda & Lubow, 1995), suggesting that the LI effect might generalize beyond standard conditioning protocols.

**Table 3:** Summary of common human latent inhibition test protocols

Task	Design	St	Snt	Phase 1		Phase 2						DV
				PE S	Masking Task (MT)	MT S	Task		O	# Snt	# Snt	
Auditory	PE	Noise	N/A	St	Identify how many times an auditory presented syllable pair is repeated.	Yes	St → O	Respond to St to prevent O	Score counter increase	1	0	Trials to criterion
	NPE			N/A								
(Adapted)	W	PE: Noise NPE: Tone		PE St			PE St → O NPE St → O			2		
Visual	PE	A shape (i.e., triangle)	A shape (i.e., square)	St	Identify repetitions of a trigram (superimposed on St in PE group).		St → O Snt → ~O			1	1	
	NPE			N/A								
Stroop H-mask	PE	H in mask	# in mask	St; Snt	Stroop task, (e.g., Stroop, 1935).					1	1	
	NPE			Snt								
Stroop BLUE–BROWN	PE	BLUE	BROWN	St							4	
	NPE			Snt								
Flanker	PE	Shape A	Shape B	St	Are letters presented *between* PE stimuli, same or different?					1	1	
	NPE			N/A								
Tsakanikos	PE	Yellow color block	Other colored blocks	St; Snt	*Variable*	No		Find words; 1 word & 3 letter strings presented per trial, in moving color blocks.	Location of word	1	3	Correct responses
	NPE			Snt								
Windows	W	PE: Yellow NPE: Green	No color	PE St	6 windows presented on screen; respond to location of O.	N/A	PE St → O NPE St → O Snt → ~O	Predict location of O	Black square	2	1	Correct responses/RT
Letter string	W	PE: S NPE: H	Letters: D, M, V, T	PE St; Snt	*Variable*	Yes	PE St → O NPE St → O Snt → O/~O	Respond to predict occurrence of X	Letter X		4	
Electrodermal	PE	Variable, e.g., Light / shape		St	N/A	N/A	St → O Snt → ~O	Differential conditioning.	Shock/ loud noise	1	0	CSR
	NPE			N/A								

PE = preexposed; NPE = non-preexposed; W = within-subjects design; St = target stimulus; Snt = nontarget stimulus; O = outcome; ~O = no outcome; MT S = masking task stimuli; DV = dependent variable; RT = reaction time; CSR = conditioned skin response

### The psychological mechanisms underpinning LI

The LI effect, as discussed thus far, is retarded responding to a stimulus that has been preexposed without consequence. As Lubow and Gewirtz (1995) observed, LI is a simple phenomenon. The empirical phenomenon is simply retarded development of responding to a stimulus that might otherwise be expected given the stimulus-outcome contingency. Despite this simplicity, there are contrasting explanations for the psychological mechanism including motoric or stimulus interference, attention, and memory. We summarize these accounts briefly before returning to the empirical effect to assess whether human tests of LI achieve the necessary controls to conclude that retarded performance is simply the result of stimulus preexposure.

Lubow et al. (1968) described the early observations of LI in terms of response interference. During the first phase, an association forms between the target stimulus and some form of response or responses that accompany the sensory habituation. This interferes with retrieval of an association between the target stimulus and the new response acquired during Phase 2. Subsequent retrieval-focused accounts have proposed that the LI effect occurs because the acquisition of an association between the target stimulus and no outcome during Phase 1 interferes with the retrieval of the association between the target stimulus and outcome acquired in Phase 2 (Bouton, 1993; Miller & Matzel, 1988; Miller & Schachtman, 1985). Such interference accounts suggest that retarded performance can be observed despite intact learning (e.g., Bouton, 1993). Supporting evidence is provided by experiments showing that LI can be disrupted by presenting the outcome in a different context (Kasprow, Catterson, Schachtman, & Miller, 1984), or extinguishing the context present in Phase 1 (Baker & Mercier, 1982; Grahame, Barnet, Gunther, & Miller, 1994; but see also Hall & Minor, 1984; Zalstein-Orda & Lubow, 1995).

Evidence supporting the interference account also suggests that LI may be context dependent, with changes in physical or temporal context between the two learning phases reducing the interference effect. For instance, the comparator hypothesis, assumes that the association formed between the stimulus and the context during Phase 1 interferes with the expression of the stimulus–outcome association in Phase 2 (Escobar, Oberling, & Miller, 2002; Miller & Matzel, 1988; Oberling, Gosselin, & Miller, 1999). A similar assumption, of context dependence, is inherent to Wagner’s (1981) standard operating procedures (SOP). SOP is a memory model in which memory resources are allocated on the basis of experience. Wagner proposed that as the preexposed stimulus is correlated with the contextual cues in Phase 1, it loses the ability to enter into association, slowing future learning (see also McLaren & Mackintosh, 2000).

**Fig. 2:**
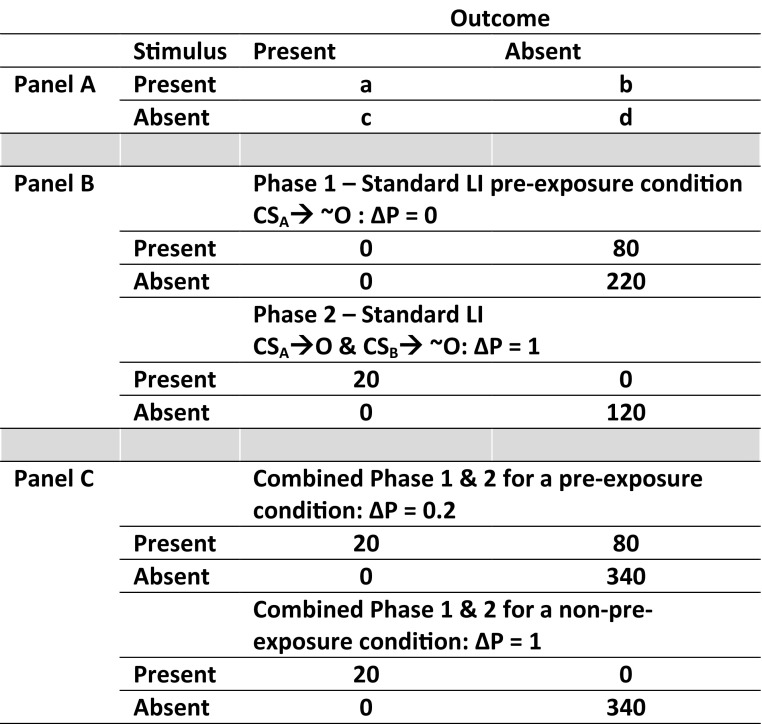
Three panels showing 2 × 2 contingency tables displaying the four possible combinations of response − outcome information. The S-O contingency is illustrated across three panels. Panel A shows a generic overview for calculating S-O contingency. S-O contingency can be viewed as a matric of four trial events, where a, b, c, and d represent the frequencies of each S-O conjunction. Contingency is determined by the difference between the likelihood of the outcome occurring in the presence of the St [a/(a + b)] with the absence of the St [c/(c + d)]. A normative model for the S-O contingency (Allan, 1980) takes the difference between these two conditional probabilities ΔP = a/(a + b) - c/(c + d). Panel B shows the S-O contingency tables for a preexposed stimulus in Phase 1 and Phase 2 of a standard LI test. Panel C shows the S-O contingency tables Phase 1 and Phase 2 combined for a preexposed and non-preexposed stimulus

The LI effect may also reflect an overall or between-sessions learning effect dependent on the aggregate contingency between S and outcome across both phases. Most LI protocols present a perfect predictive relation between S and outcome in Phase 2. However, integrating the Phase 1 contingency experience of the S followed by no outcome, with the Phase 2 contingency, of a S followed by an outcome, will reduce the overall S–outcome (S-O) contingency substantively (for further discussion, see Baker, Murphy, & Vallée-Tourangeau, 1996; Le Pelley & Schmidt-Hansen, 2010; Schmidt-Hansen & Le Pelley, 2012). This is illustrated in Fig. 2 for an LI task including 80 preexposure trials in Phase 1 (cell *b*), interspersed with intertrial intervals (cell *d*) followed by Phase 2 training. Where Phase 1 and Phase 2 are considered separately, the CS-O contingency (ΔP) is zero in Phase 1 and one in Phase 2. Where these two phases are combined, the overall CS-O contingency in a non-preexposed condition remains one but is reduced to 0.2 in the preexposed condition. Therefore, the two phases provide evidence consistent with a reduced overall relation.

So far, the explanations have centered on competing responses or interfering or contrasting memory traces. Another approach invokes attentional processes. Experience with a stimulus may result in lowering attention directed to that stimulus, reallocating processing resources away from the preexposed stimulus, resulting in slowed learning (e.g., Lubow, 1989; Mackintosh, 1975; Pearce & Hall, 1980). Hybrid accounts of stimulus associability modulation have been developed, recognizing that both previous relative predictive validity and repeated presentations can influence stimulus associability (Le Pelley, 2004; Pearce & Mackintosh, 2010).

There are different accounts of the psychological mechanisms underpinning the LI effect. LI can be explained in terms of interference, attention, or memory. Perhaps because of the evidence, there is no consensus for a mechanism underlying LI. Further, and of direct relevance to this review, it is possible that different psychological mechanisms are relevant for different demonstrations of LI. Here, we address issues related to the methodology for assessing human LI. We further consider whether the different methods that have been employed can discriminate between the different psychological accounts.

## Overview for tests of human LI

In a selective review of the literature, we identified 59 studies of adult human LI that either focus on general mechanisms or individual differences in LI. The majority (*n* = 52) report some form of retardation in performance to the preexposed stimulus. There is no denying that preexposing a stimulus can retard subsequent responding to that stimulus when it is paired with an outcome. However, while LI is descriptively simple, this simplicity has proved to be a methodological challenge. Few of the human experiments we reviewed simply preexposed a stimulus and then measured changes in performance. Human participants tend to require explanation and instruction even regarding a simple stimulus exposure experience. Such deviations from the simplest design introduce confounds, creating alternate explanations for the apparent retardation. This raises the question of whether any of the existing human LI research provides the necessary experimental controls to support the conclusion that the LI effect measured is caused simply by preexposure to a stimulus. We review the extensive range of methods used to test human LI with the aim of assessing whether human tests of LI have been able to simply measure the effect of stimulus preexposure on performance.

The studies we reviewed used 11 different experimental protocols. Further details of the eight more common protocols are summarized in Table 3. The earliest of these protocols was the auditory task that was run, almost universally, as a between-subjects task (e.g., Allan et al., 1995; Baruch et al., 1988a, 1988b; Braunstein-Bercovitz & Lubow 2004; Ginton et al., 1975; Gray, Snowden, Peoples, Hemsley & Gray, 2003; Kumari et al., 1999; Lubow, Weiner, Schlossberg, & Baruch, 1987; Lubow et al., 1992; Serra, Jones, Toone & Gray, 2001; Williams et al., 1996; Williams et al., 1998; Wuthrich & Bates, 2001), with the exception of two within-subjects designs (Gray, Pilowsky, Gray, & Kerwin, 1995; Gray et al., 2003). The visual task minimally adapted the auditory task and was widely used as a between-subjects test of LI (e.g., De La Casa, Ruiz, & Lubow, 1993; Gray et al., 2001; Gray et al., 2002; Lipp, 1999; Lipp, Siddle, & Arnold, 1994; Lubow et al., 1992; Rascle et al., 2001; Swerdlow et al., 1996; Zalstein-Orda et al., 1995). In addition to the auditory and visual tasks, a number of studies have used an electrodermal response procedure (e.g., Booth, Siddle, & Bond, 1989; Björkstrand, 1990; Lipp et al., 1994; Lipp & Vaitl, 1992; Siddle, Remington, & Churchill, 1985; Vaitl et al., 2002).

There have been several infrequently used adaptations of the visual task. Williams et al. (1996) developed a variant of the visual task that employed a form of Stroop task (see also Williams et al., 1997). Braunstein-Bercovitz and Lubow adapted the LI visual task to include a flanker masking task in Phase 1 (e.g., Braunstein-Bercovitz, 2000; Braunstein-Bercovitz & Lubow 1998a, b). De La Casa and Lubow adapted the visual task further to embed it within a prediction task requiring participants to predict where on the screen the outcome would occur, we refer to this task as the windows task (e.g., De La Casa, 2002; De La Casa & Lubow, 2001). Tsakanikos and colleagues provided a different visual LI protocol, requiring participants to find letter strings in moving color blocks (Tsakanikos & Reed, 2004; Tsakanikos, Sverdrup-Thygenson, & Reed, 2003). Most recently, Evans and colleagues embedded a visual LI task within a simple letter prediction task, in which participants have to learn which letters predict the occurrence of a letter *X* (Evans, Gray, & Snowden, 2007). This task has been widely used to test individual differences in LI (e.g., Granger et al., 2016; Gal et al., 2009; Granger, Prados, & Young, 2012; Schmidt-Hansen & Honey, 2014; Schmidt-Hansen, Killcross, & Honey, 2009; Shrira & Kaplan 2009).

As shown in Table 3, these protocols have differed in their use of between-subjects and within-subjects designs, the types of stimuli used, the task employed in Phase 1, the task employed in Phase 2, the ratio of target to nontarget trials in Phase 2, and the dependent variable. This range creates opportunities for variation in methods to influence experimental findings. We assessed whether the protocols were sufficiently simple to provide an unambiguous assessment of LI. Our discussion focuses upon three potential confounds: (a) the general task procedure during preexposure, (b) the multiple control cues and their effects on relative novelty, and (c) the response requirements and the dependent measures. Before addressing these, we set out a general review of variations in key LI parameters.

### Variation in key parameters

Almost all of the studies reviewed involved a two-phase design with one stimulus (St) presented without consequence in Phase 1 prior to the same stimulus predicting an outcome in Phase 2 (see Lipp, 1999, for exception). In all of the studies, a comparison was made of Phase 2 performance for a preexposed (PE) St and a non-preexposed (NPE) St, both of which predict an outcome in Phase 2. Traditionally, tests of LI are conducted with a between-subjects’ design, with one group preexposed to St and a control group not receiving preexposure (see Table 3; auditory, visual, Stroop, flanker, Tsakanikos, and electrodermal tasks). Recent studies have adapted the protocol to allow a within-subjects’ test (see Table 3; windows and letter string tasks). Where studies have adopted a within-subjects’ design, participants are presented with two target stimuli in Phase 2, a PE St and an NPE St, both of which predict the outcome. LI has been observed with both between-subjects and within-subjects designs.

Many tests of LI also include a masking task (see Table 3; auditory, visual, Stroop, and flanker tasks). Lubow and Gewirtz (1995) argued that a masking task in Phase 1 is necessary to engage the participants’ attention in controlled information processing and, in consequence, leave the preexposed stimulus to automatic processing (Schneider & Shiffrin, 1977; Shiffrin & Schneider, 1977). This justification depends on the theoretical interpretation that LI is an automatic attentional effect. Furthermore, Lubow and Gerwitz (1995) argued that the Phase 1 masking task is necessary to reduce demand characteristics; that is, human participants may have an enhanced allocation of attention to cues during preexposure because they are participating in an experiment and may assume that all stimuli have a level of experimental relevance. Thus, the masking task is employed to reduce the likelihood of participants maintaining artificially high levels of attention to the preexposed stimuli. Both of these justifications for a masking task have been critiqued (e.g., Escobar, Arcediano, & Miller, 2003; Granger et al., 2016; Schmidt-Hansen & Le Pelley, 2012). Recent studies including the letter string task indicate that LI effects can be observed without a masking task (Evans et al., 2007; Gal et al., 2009; Granger et al., 2012; Granger et al., 2016; Schmidt-Hansen et al., 2009; Shrira & Kaplan, 2009).

As LI refers to the effect of preexposure on subsequent performance, the amount of preexposure may be expected to be an important factor contributing to the magnitude of the LI effect. While we may expect more preexposure trials to result in a stronger LI effect, this is not necessarily the case. The number of preexposure trials necessary to elicit a robust LI effect may depend on the task and stimuli. Different experiments have used different levels of preexposure. For instance, while it is common to have 30 preexposure trials in the auditory task (e.g., Baruch et al., 1988a, 1988b; Lubow et al., 1992; Lubow et al., 1987; Swerdlow et al., 1996), 80 preexposure trials are included routinely in the visual version of the task (e.g., Gray et al., 2001; Gray et al., 2002; Lubow et al., 1992). LI only appears reduced when the number of stimulus preexposures drops below 10 (e.g., Allan et al., 1995; Williams et al., 1996, 1998).

Most protocols include a discrimination task in Phase 2, such that a target stimulus (St) predicts the outcome while other nontarget stimuli (Snt) do not (see Table 3; visual, Stroop, flanker, Tsakanikos, windows, letter string, and electrodermal tasks). This design contrasts with the original auditory task, used by Ginton et al. (1975), which did not include a discrimination; participants simply learnt that the target stimulus predicted a paired outcome. While most protocols have included both a St and Snt, there has been variability in the preexposure of these stimuli. For example, in Lubow’s original design only the St was preexposed. A similar approach has been adopted in the auditory, visual, flanker, and windows tasks. Other protocols pre-expose both the St and Snt in Phase 1 (see Table 3; Stroop, Tsakanikos, and letter string tasks). We discuss below how this difference influences the relative novelty of the NPE St.

### Are studies of human LI measuring only the effect of stimulus preexposure?

If LI is only an effect of retarded performance following stimulus preexposure, any test has to be able to demonstrate that retarded performance is the result of stimulus pre-exposure and cannot be explained by other factors. We argue that at least three of the parameters varying between tests of human LI have the potential to introduce substantive confounds: (1) the task adopted in Phase 1; (2) the comparison between preexposed (PE) and non-preexposed (NPE) target stimuli (St), which we refer to as relative novelty effects; and (3) the response requirements across the test and the dependent variables used to assess performance in Phase 2.

#### The Phase 1 task

Most studies of human LI have included some strategy to ensure participant engagement during the first phase of the task. We have identified three different approaches to engaging participants during Phase 1: (1) a making task using stimuli distinct from the Phase 2 learning task, (2) consistent instructions across Phase 1 and 2, and (3) a task, independent from the Phase 2 learning task, but directly involving the stimuli to be presented in Phase 2. These second two approaches include a task to engage participants’ attention during Phase 1, but do not introduce additional stimuli. The use of these three approaches in the Phase 1 tasks is summarized in Table 3. Both the first and second approaches, and some variants of the third approach, create potential confounds for assessing the effect of preexposure on subsequent task performance, as outlined below.

##### Masking task

The inclusion of a separate and distinct masking task has been the most common approach to a Phase 1 task, used by 39 of the studies we reviewed. As outlined in Table 3, the auditory, adapted auditory, visual, Stroop, and flanker tasks have included a distinct masking task. The masking task is completed in Phase 1, and the masking task stimuli are usually also present in Phase 2. For instance, with the auditory task, the masking task consists of the presentation of an auditory syllable list, and participants are required to count the number of times that a specific syllable occurs (e.g., Ginton et al., 1975). In the preexposure (PE) group, the St, a white noise, is interspersed randomly between syllables. In Phase 2, the syllable list is repeated with white noise interspersed between syllables for both the PE and NPE groups.

This approach bears some similarity to animal experiments, such as Rescorla (1971), which also included a simultaneous secondary task. In these animal experiments, that might be considered the “gold standard” of LI, the subjects are engaged in an ongoing and unrelated behavior (i.e., lever pressing for food). Superimposed on this behavior is the multiple presentation of an unrelated discrete auditory stimulus (CSt). During Phase 2, the ability to learn about the CSt as a predictor of an outcome is assessed. Rescorla found retarded acquisition of this relation; however, notice that the CSt is presented within a context involving actions (lever pressing) and outcomes (food) that bear no relation to the CSt–outcome association acquired in Phase 2.

Both human and animal tests of LI have included masking tasks. However, not all human experiments have included a masking task and the variability in methods leads to the possibility that different tasks are measuring different effects. Further, there are several factors associated with the human masking task that may introduce confounds and provide alternative explanations for the apparent LI effect. Specifically, holding the masking task stimuli constant between Phases 1 and 2 provides preexposure to the masking task stimuli and the context, effectively diminishing the novelty of these stimuli and thus accentuating the relative novelty of the NPE St. We return to discuss this effect of relative novelty in detail below.

By incorporating masking task stimuli in Phase 1, the protocol, involves an experimental requirement to shift attention away from the preexposed stimuli (see Escobar et al., 2003, for further discussion). This manipulation may create a negative priming effect (e.g., Graham & McLaren, 1998; Tipper, 1985, 2001). That is, during the preexposure phase, the masking task stimuli *are* target stimuli and the PE St is effectively a distractor to be ignored. This requirement, to ignore the St, may suppress the internal representation of the stimuli, reducing subsequent ability to recognize these stimuli (Tipper, 1985, 2001). In the second phase of the task, this relationship is reversed, with the requirement to ignore the previously relevant masking task stimuli and attend to the previously irrelevant PE St (Escobar et al., 2003; see also Tsakanikos et al., 2003). Thus, effects of preexposure observed in LI protocols including a masking task might be caused by negative priming and reflect a delay in *shifting* attention from masking task stimuli to the PE St.

Is this a problem? One could argue that outside of the laboratory, most scenarios that reflect LI also include other stimuli to which attention is directed. It is, however, important to be able to recognize when an experiment has measured a simple effect of preexposure compared with an effect of switching attention. Where requirements to switch attention are driving reduced performance, we should be considering the relation to other similar effects, such as those of extradimensional and intradimensional shifting (e.g., Slamecka, 1968). Importantly, while many experiments have included masking tasks that introduce such a requirement to shift attention, many others (discussed below) report retarded performance with preexposed stimuli in the absence of this requirement to shift attention. It is unclear whether these experiments are measuring the same effect.

##### Same instructions

The second approach to engaging participants’ attention in Phase 1, employed with the letter string protocol and Tsakanikos task, has been to use the same instructions during Phases 1 and 2. This approach was used by eight of the studies that we reviewed. Here, during Phase 1, the outcome, to be predicted, is presented so that it is not predicted by any stimulus reliably (Gal et al., 2009; Shrira & Kaplan, 2009) or is not presented at all (Escobar et al., 2003; Evans et al., 2007; Granger et al., 2016; Granger et al., 2012; Schmidt-Hansen et al., 2009; Tsakanikos et al., 2003). Efforts to remove the masking task from tests of human LI have improved the experimental design by simplifying the protocol. However, providing experience of the preexposed St being an unreliable predictor of the outcome, or a less reliable predictor of the outcome than other stimuli, can change the protocol from a direct test of LI (preexposure to a stimulus without consequence), and instead set up a test of learned irrelevance (LIRR; Bonardi & Ong, 2003), which is distinct from LI. For instance, in Phase 1, Shrira and Kaplan (2009) asked participants to respond when they thought the outcome would occur. On successive trials, participants were shown a St intermixed with other distractor stimuli. The outcome was presented, but was not predicted by any stimulus reliably. Experiments adopting such a procedure for Phase 1 provide explicit opportunity for participants to learn that the St is an *unreliable* predictor of the outcome. This learning alone, independent from simple stimulus preexposure, would be expected to retard subsequent performance (Baker & Mackintosh, 1979; Bennett, Wills, Oakeshott, & Mackintosh, 2000; Le Pelley & Schmidt-Hansen, 2010; Mackintosh, 1973; Schmidt-Hansen & Le Pelley, 2012). For instance, unreliable or invalid cues enhance the relative validity of other trained cues or even the context (e.g., Murphy, Baker, & Fouquet, 2001). Thus, experiments presenting *any* outcome during Phase 1 are inherently ambiguous, as retarded performance may reflect either stimulus preexposure, a relative validity effect, LIRR, or a combination (see also Baker & Mackintosh, 1979; Le Pelley & Schmidt-Hansen, 2010).

Where participants are instructed to expect the occurrence of an outcome at the outset of the task, they may be encouraged explicitly or implicitly to encode the *absence* of the outcome during Phase 1 as a meaningful event. Participants may thus be likely to integrate their experience of an association between St and no outcome from Phase 1 into Phase 2 to generate an overall perception of a reduced predictive status of the PE St compared to the NPE St (Schmidt-Hansen & Le Pelley, 2012). Consider, for example, Escobar et al. (2003), who removed the distinction between Phase 1 and 2, requiring participants simply to observe a succession of stimuli before making judgments about the predictiveness of the PE St and NPE St. Participants were presented with 10 presentations of an St followed by no outcome, after which the training schedule continued without interruption to one presentation of the same St followed by an outcome. In this protocol, integrating the two phases alters the programmed contingency between PE St and NPE St and the paired outcome, generating a stimulus–outcome (S-O) contingency of 1/11 (.09) for the PE St relative to 1/1 (1) for the NPE St (for further discussion, see Le Pelley & Schmidt-Hansen, 2010; Schmidt-Hansen & Le Pelley, 2012). Differences in the rates of responding to the PE and NPE St in Phase 2 can be attributed here either to preexposure to the St or sensitivity to the overall contingency (e.g., Baker et al., 1996).

Informing participants about the outcome may encourage them to learn a stimulus–no outcome association in Phase 1 and integrate this with their Phase 2 learning to reduce the overall experienced contingency. However, removing information about the outcome provides no guarantee that participants are not integrating the trial experience relevant to the experienced contingencies across Phase 1 and Phase 2. Thus, retarded performance with a preexposed St may be a result of reduced S-O contingency. Attention-based accounts of LI assume that this is not the case because participants have no knowledge of the outcome in Phase 1 and should not be able to acquire an association between the stimulus and no outcome.

The contingency between stimulus and outcome is only one factor influencing learning; other factors include surprise (Rescorla & Wagner, 1972) and recency (Pineño & Miller, 2005). However, there is considerable evidence that animals (Baker, 1976; Baker & Mackintosh 1979) and humans can learn contingencies and that they can be learnt across phases and across sessions (e.g., Baker et al., 1996; Baker, Murphy, & Mehta, 2003). Importantly for this review, the LI literature has so far overlooked the potential influence of contingency. Existing tests of LI do not rule out the influence of contingency, creating ambiguity in terms of the psychological mechanisms underlying the LI effect.

The role of contingency learning in the LI effect could be tested. The standard LI test can be set out in terms of contingency, as illustrated in Fig. 2. In current tests, the S-O contingency differs between the PE St and NPE St. Participants who are preexposed to the St experience a much lower S-O contingency (Schmidt-Hansen & Le Pelley, 2012). One way to isolate the influence of contingency on the effect of stimulus preexposure would be to minimize the difference in S-O contingency between preexposed and non-preexposed conditions. If LI can be explained in terms of contingency, matching the S-O contingency for PE and NPE conditions should eliminate the difference in performance between the conditions. Matching the contingency for PE and NPE conditions can be achieved by changing the ratios of St and Snt trials. This is possible because the overall contingency can be changed by altering any individual component of the S-O contingency. For example, to match the S-O contingency between the PE and NPE conditions, the probability of the outcome occurring in the absence of the stimulus through Phase 2 can be manipulated to decrease the S-O contingency in the NPE condition. Alternatively, S-O contingency can be held constant while preexposure is manipulated. For instance, the standard LI protocol presents a block of stimulus–no outcome (cell *b*) trials at the start of the task, before introducing stimulus–outcome (cell *a*) trials. If preexposure has an effect, over and above contingency, the effect should be eliminated when cell *b* trials are intermixed with cell *a* trials in a single phase.

##### Tasks involving the to-be-presented stimuli

The third approach to engaging participants during Phase 1 has been to instruct participants to engage with the stimuli to be presented in Phase 2 by tracking the occurrence of a specific stimuli (e.g., Granger et al., 2016), counting the number of stimuli that appear (e.g., Shrira & Kaplan, 2009), making a judgment about the stimuli (e.g., Tsakanikos & Reed, 2004), or reading the stimuli out loud (e.g., Granger et al., 2016). This third approach has been used with the letter string protocol and Tsakanikos task (see Table 3) and was employed in five of the studies we reviewed (De La Casa & Lubow 2001; Granger et al., 2016; Schmidt-Hansen & Honey, 2014; Shrira & Kaplan, 2009; Tsakanikos & Reed 2004). While this approach may avoid many of the concerns discussed above, requiring participants to track *specific* stimuli, such as counting the number of times that a specific nontarget stimulus occurs, might shift the participant’s attention away from the St, creating similar negative priming effects to those discussed above.

##### No Phase 1 task

Many of the studies involving an automatic electrodermal response have been conducted without a masking task. In this sense, these tasks provide a relatively unambiguous test of human LI, and it is worth noting that several of these experiments report evidence of LI (Björkstrand, 1990; Lipp et al., 1992; Siddle et al., 1985). However, while we have included studies involving conditioned skin conductance responses in this review, others have raised concerns that these studies report a retardation of autonomic responses that can be attributed to habituation to the preexposed stimulus (Escobar et al., 2003). Habituation occurs when repeated presentation of a single stimulus produces a reduction in responding to the stimulus. Retardation of the acquisition of skin conductance may reflect a peripheral process of habituation, which is not necessarily an associative process and has been dissociated from latent inhibition (Hall, 1991).

#### Summary

In terms of the Phase 1 task, only simple tasks, involving the to-be-presented stimuli (e.g., Granger et al., 2016; Shrira & Kaplan, 2009) and tests including no Phase 1 task have met the requirements of simplicity and avoided alternate explanations for retarded performance. The corollary is that many tests of human LI are ambiguous, and observation of retarded performance with the preexposed target stimulus is open to alternative interpretations.

Interestingly, where different approaches to the Phase 1 task have been employed within the same protocol, the effect on task performance is minimal. For instance, within the letter string and Tsakanikos tasks, two different Phase 1 tasks have been employed: (a) using the same instructions in both phases and hence accentuating the likelihood that participants integrate the Phase 1 and Phase 2 contingencies; or (b) using a task involving stimuli to be presented in Phase 2, minimizing the likelihood of integrating Phase 1 and Phase 2 contingencies. An LI effect was observed in both conditions with the Tsakanikos task (Tsakanikos & Reed, 2004; Tsakanikos et al., 2003) and the influence of the Phase 1 task in the letter string protocol also appears to be similarly minimal (Granger et al., 2016; Shrira & Kaplan, 2009). While the Phase 1 task thus appears to have limited impact on the empirical LI effect, different Phase 1 tasks introduce alternate mechanisms for producing this effect, and it is not clear that the effect of retarded performance with a preexposed stimulus is caused by the same underlying mechanism or, indeed, whether retarded performance can, in all experiments, be attributed exclusively to the preexposure of a stimulus.

#### Relative novelty

Mismatches in stimulus novelty facilitate learning (e.g., Balaz, Capra, Kasprow, & Miller, 1982; Lubow, Schnur, & Rifkin, 1976; Schmajuk, Lam, & Gray, 1996). A mismatch in novelty can be created by presenting a novel stimulus in a preexposed context, or by presenting a preexposed stimulus in a novel context. In contrast, learning is expected to be impaired when a preexposed stimulus is presented in a preexposed context. Tests of LI attempt to measure the degree of performance impairment induced by such preexposure. LI is measured by comparing performance impairment with a preexposed stimulus (PE St) to performance with a non-preexposed stimulus (NPE St). The NPE St is usually a novel stimulus being presented in a preexposed context. Thus, for the NPE St there is a mismatch in novelty which is expected to facilitate performance. Importantly, this facilitation effect is more substantive than presenting a novel stimulus in a novel context (e.g., Pearce & Hall, 1980; Schmajuk et al., 1996). Therefore, many tests of LI confound effects of preexposure (of the PE St) and novelty (with the NPE St). That is, rather than simply measuring a preexposure effect, they also measure a relative novelty effect.

The separate contributions of preexposure and relative novelty are shown in Table 4. In tests of LI, performance with the PE and NPE stimuli are compared. These stimuli are shown in Table 4 as stimulus X (PE St) and stimulus Y (NPE St). Focusing on a comparison of PE and NPE St, Conditions 1 and 2 are treated as providing the same test of LI; both provide an opportunity to compare a PE and NPE St. However, by using non-preexposed distractors (Snt) in Condition 1, the NPE St (Y) is not relatively novel. In comparison, in Condition 2, as in most tests of LI, the distractor stimuli (Snt) are preexposed, so that the NPE St (Y) is relatively novel, in addition to not being preexposed.

**Table 4:** Tests of latent inhibition with and without relative novelty effects

Condition	Phase 1: Preexposure phase	Phase 2: Test phase
Condition 1: No relative novelty effect	A, B, C, X	X → O; Y → O; D, E, F
Condition 2: Relative novelty effect	A, B, C, X	X → O; Y → O; A, B, C

A, B, C, D, E, F, X and Y represent stimuli; O represents an outcome

The relative novelty confound exists in all experiments that have maintained the masking task stimuli between Phases 1 and 2 (e.g., in the auditory, visual, Stroop, and flanker tasks). When the NPE St is introduced at the start of Phase 2, it is novel relative to all other stimuli (the masking task stimuli, the contextual stimuli, etc.) Unfortunately, the confound of relative novelty has also influenced LI in the absence of a masking task. Most tests of LI include a discrimination task in Phase 2, with participants required to discriminate a target stimulus from one or several nontarget stimuli (see Table 3). With a discrimination task, preexposure to the Snt accentuates the relative novelty of the NPE St, by ensuring that the context in which the novel NPE St is presented is preexposed. As summarized in Table 3, the Stroop H-mask task, Tsakanikos task, and letter string task all preexpose the Snt. For example, in the letter string protocol, at the start of Phase 2, the PE St and all of the Snts have been preexposed. In contrast, the NPE St is the *only* novel stimulus.

To our knowledge, no study has manipulated the relative novelty of the NPE St systematically. However, both Tsakanikos and Reed (2004) and Pineño, De La Casa, Lubow, and Miller (2006) manipulated preexposure of the Snt within a single study, changing the relative novelty of the NPE St. Further, while most letter string protocols preexpose the Snt (e.g., Evans et al., 2007; Granger et al. 2016; Schmidt-Hansen et al., 2009; Shrira & Kaplan 2009), Gal et al. (2009) changed the Snt between Phases 1 and 2 again, changing the novelty of the NPE St. While the first two studies suggest that relative novelty contributes to the LI effect (Pineño et al., 2006; Tsakanikos & Reed, 2004), the third does not (Gal et al., 2009). Pineño et al. (2006) observed relatively impaired performance with a preexposed stimulus across a range of experimental manipulations using a between-subjects design, but this was not observed when the Snt was changed between Phases 1 and 2, reducing the relative novelty of the NPE St. Tsakanikos and Reed (2004) reported a similar effect. However, using the letter string task, Gal et al. (2009) changed Snt between Phases 1 and 2, again reducing the relative novelty of the NPE St, and found a strong LI effect, contrasting the findings of the other two experiments.

Future experiments can and should control relative novelty. The letter string task can be adapted to ensure the NPE St is not relatively more novel than other stimuli. This is shown in Table 4. Controlling relative novelty is essential to provide an unambiguous measure of the effect of stimulus preexposure.

#### Response requirements and dependent variables

##### Response requirements

The ratio of target (St) to nontarget (Snt) trials reflects the proportion of trials on which participants are expected to respond. We may expect the ratio of response to no response trials to affect the response accuracy and the measured LI effect (e.g., Donkers & Van Boxtel, 2004; Kiehl, Liddle, & Hopfinger, 2000). However, while the ratio of St to Snt trials has varied between experiments, no studies have assessed the effect that varying this ratio has on the strength of the LI effect systematically. Looking at variations in the ratio of response requirements between experiments using the same protocol, the ratio does not appear to have a substantive effect.

More generally, the response requirements are not manipulated across tests of LI (for exceptions, see Msetfi et al., 2017; Nelson & Sanjuan, 2006). Almost all of the tests of human LI that we reviewed here required participants not to respond to the St in Phase 1 before learning to respond to the St in Phase 2. Early explanations of LI focused on response effects. Lubow et al. (1968) suggested that the stimulus–no response association acquired in Phase 1 interfered with retrieval of the stimulus–response association acquired in Phase 2. While this was one of the very earliest explanations for LI, tests of human LI have not manipulated the response requirements to dissociate the contribution of response learning. Thus, the current human LI literature cannot rule out the possibility that retarded performance following preexposure is an entirely behavioral effect.

Exploring the contribution that acquisition processes play for the response phase might be achieved by manipulating the Phase 1 and Phase 2 response requirements, for instance, requiring participants to respond to stimuli predicting no outcome in Phase 2 or setting up a task in Phase 1 that required participants to respond to the preexposed stimuli. This would allow stimulus–response and stimulus–outcome associations to be manipulated between Phase 1 and Phase 2 independently.

##### Dependent variables

While response requirements have not been manipulated, several different dependent measures have been used. In the introduction, we considered the benefit of measuring the change in a suppression ratio over multiple trials within animal studies of LI (Rescorla, 1971). This approach provided a more detailed dependent measure than the trials-to-criterion measure used previously and captured a comparison between the rate of responding in the presence of the St relative to responding in the absence of the St. Human tests of LI have used a variety of different dependent measures, including trials to criterion, prediction of the outcome likelihood, the total number of correct responses, mean or median response time, or conditioned skin conductance response. As we argue here, none of these dependent measures provides the same level of detail as described by Rescorla (1971), and this limits our ability to assess whether preexposure retards performance. Further, the range of dependent measures limits our ability to make meaningful comparisons between protocols.

##### Trials to criterion

The trials-to-criterion measure has been used widely, being adopted in the auditory, visual, Stroop, flanker, and Tsakanikos tasks (see Table 3). This dependent measure is the number of trials that it takes a participant to reach a certain criterion of accuracy in Phase 2. It is a single dependent measure, combining response accuracy to the St and Snt. This facilitates comparison between the preexposure and non-preexposure conditions as well as assessment of individual differences. However, with such simplification, important details about response accuracy are lost.

Take, for example, a participant who takes 16 trials to reach criterion. It is not possible to tell whether this single number reflects response accuracy in the presence of St or Snt. The criterion usually adopted has been five consecutive correct responses to the St with no responses to the Snt. Because the St is preexposed, it may be assumed that 16 trials to criterion reflects slow acquisition of response accuracy to the St, prompting the conclusion that it took 16 trials for the participant to start responding to the St correctly. However, we cannot tell when accurate responding to the Snt was established, and it may be that it takes a similar amount of training to establish correct responding to the Snt. Alternatively, correct responding to the Snt might have been established early in training, before the criterion was reached. In this respect, the trials-to-criterion measure is uninformative about the number of trials taken to achieve correct responding in the presence of the Snt.

Another problem is that 16 trials to criterion could reflect the number of trials necessary to achieve correct responding in the presence of the Snt. That is, it is possible for the participant to have acquired correct responding to St rapidly, but continue making errors, incorrectly responding to the Snt. In this way, the trials-to-criterion measure might also be described as uninformative about the number of trials taken to achieve correct responding to St (Msetfi et al., 2017).

Indeed, it is not possible from the single measure to identify whether differences in trials to criterion reflect acquisition of correct responses to St or Snt. This is important because we assume that preexposure to the St slows the acquisition of the correct response to the PE St, but the trials-to-criterion measure does not assess this explicitly. It gives a measure of the overall response accuracy following preexposure, which may reflect an effect of preexposure on acquisition of response accuracy to the PE St or the Snt. In this respect, the measure is entirely ambiguous.

With this in mind, one may argue that there is an advantage in studies that consider only response accuracy in the presence of St (e.g., Gray et al., 2002; Gray et al., 2003; Gray et al., 2001). By ignoring response accuracy to Snt, analysis of response accuracy to St is unambiguous about the accuracy of responding on St trials. However, unfortunately, there are problems here, too. In a design where participants should respond in the presence of St and withhold responding in the presence of Snt (as seen in all of the LI protocols discussed in this review), the participant who responds in the presence of St selectively and the participant who responds on every single trial indiscriminately could have the same trials-to-criterion score. As such, this measure is entirely uninformative about performance.

##### Response accuracy and reaction time

Recent experiments (i.e., since 2001) have moved away from the trials-to-criterion measure, measuring the number of correct responses (e.g., Tsakanikos and letter string tasks) or reaction time (e.g., windows and letter string tasks). While there are many advantages to these measures, they have often been used without any consideration of response accuracy on Snt trials. For instance, Evans et al. (2007) measured the number of correct responses in the letter string task, counting the total number of times that participants responded in the presence of St. This did not incorporate a measure of response accuracy in the presence of Snt. As such, the data cannot discriminate between selective and indiscriminate responding.

Measuring reaction time provides an opportunity for trial-by-trial analysis, to assess how changes in the *rate of responding* emerge over Phase 2. However, most studies of LI have collapsed the Phase 2 data to provide a single dependent measure, for instance taking the mean or median reaction time across the whole of the Phase 2 training. This precludes analysis of differences in the rate of change in responding. As LI is described as retarded performance following preexposure, analysis of the rate of change in responding should be central to understanding LI.

## Summary

Latent inhibition should be a simple effect of retarded task performance following experience with a stimulus in the absence of a consequence. Despite this simplicity, few experiments carried out with human participants have used sufficiently simple protocols and included sufficient experimental controls, in order to constrain the source of the retarded performance to the effects of pre-exposure. Although there are more than 59 experiments testing human LI, none provides a purely unambiguous assessment of the effect of stimulus preexposure on performance. Future research needs to return to a simpler experimental design, ensuring that alternate explanations for retarded performance are not possible. While our review has highlighted several potential confounding factors, their effect on LI has not been explored systematically. Without this systematic exploration, we are unable to rule out several alternate explanations for apparent retardation following preexposure, as we summarize below. We recommend the following as a set of basic requirements for future tests of LI.

### A simple Phase 1 task

From a practical perspective, the nature of the Phase 1 task does not appear to have a dramatic influence on the effect of preexposure. However, there are complications in interpreting the effect that most Phase 1 tasks have on Phase 2 performance. Many Phase 1 tasks have altered the LI design to a learned irrelevance design (Le Pelley & Schmidt-Hansen, 2010; Schmidt-Hansen & Le Pelley, 2012), introduced possible negative priming (Escobar et al., 2003), or changed the task to one of contingency learning (for further discussion see, Le Pelley & Schmidt-Hansen, 2010; Msetfi et al., 2017; Schmidt-Hansen & Le Pelley, 2012). Very few studies have avoided these confounds, which leads to the disappointing caution that most studies reporting human LI are ambiguous in method and theoretical interpretation.

A few recent experiments have shown that simply requiring participants to engage with the Phase 2 stimuli through Phase 1 does not disrupt LI (Granger et al., 2016; Schmidt-Hansen & Honey, 2014; Shrira & Kaplan, 2009; Tsakanikos & Reed, 2004). Looking toward future research, this approach provides an opportunity to test LI without biasing a participant’s attention toward or away from any particular stimuli, limiting alternate explanation for the effect of preexposure.

### Dissociate preexposure from relative novelty

Most tests assess LI as the difference in performance with a PE St and an NPE St. However, the novel stimulus is usually presented in a preexposed context, facilitating learning with the novel stimulus (Pearce & Hall, 1980; Schmajuk et al., 1996). Thus, the reported LI effect is actually a combination of preexposure and relative novelty effects in which the relative contribution of each effect is unknown. Future research needs to manipulate the relative novelty of the NPE St to dissociate the contributions of preexposure and novelty to the LI effect. Future assessments of LI can isolate the effect of preexposure by including both preexposed and non-preexposed nontarget stimuli to ensure that, at the start of Phase 2, the non-preexposed target stimulus is not relatively novel.

### Measure the rate of change in behavior

Despite LI being described as a slow change in responding following preexposure, few studies have analyzed the rate of change in responding following preexposure, limiting our ability to understand how preexposure influences performance. Future research would benefit from trial-by-trial analysis to explore these interactions further. Also, using single measures of LI oversimplifies the interpretation of the effect. Instead, future studies need to report response accuracy across all trial types and analyze how this changes over trials of training.

### Conclusion

As Lubow states, LI is a simple phenomenon (Lubow & Gerwitz, 1995). Elegantly designed animal studies have provided empirical clarity that has allowed the LI literature to play a key role in the development of modern learning theory (e.g., Kruschke, 2001; Le Pelley, 2004; Mackintosh, 1975; McLaren, Kaye, & Mackintosh, 1989; McLaren & Mackintosh, 2000; Pearce & Hall, 1980; Pearce & Mackintosh, 2010; Wagner, 1981). Animal studies of LI are widely employed to examine disruptions in cognitive and behavioral flexibility, yet for this LI research to have the translational impact in human research, informing our understanding of diversity in cognitive and behavioral flexibility, tests of LI in humans require significantly more empirical control. Achieving simplicity within human studies has been challenging. Despite the extensive use of LI in animal models of human mental health, we have yet to see conclusive evidence of human LI, let alone a comprehensive understanding of the psychological mechanisms underpinning the effect.
